# Spontaneous Reversal of Vitiligo, a Rare Phenomenon Reported in a Case in Saudi Arabia with an Insight into Metabolic Biochemical Derangements

**DOI:** 10.3390/medicina59030427

**Published:** 2023-02-22

**Authors:** Ayoub Ali Alshaikh, Rishi Kumar Bharti

**Affiliations:** Department of Family and Community Medicine, King Khalid University, Abha 62585, Saudi Arabia

**Keywords:** herbal remedies, reversal, vitiligo, biochemical mechanisms, treatment, curcumin

## Abstract

*Background and Objectives:* Vitiligo is a skin disorder characterized by hypopigmented macules occurring due to melanocyte destruction. An interplay of several biochemical mechanisms has been proposed to explain the etiopathogenesis of vitiligo, such as genetic, autoimmune responses, generation of inflammatory mediators, oxidative stress, and melanocyte detachment mechanisms. There is no cure for vitiligo; however, pharmacological treatment measures (cosmetic camouflage creams, steroids, psoralen and ultraviolet A (PUVA) therapy, narrowband UVB) are available, but they could have certain side effects. We reported an interesting case of vitiligo in Saudi Arabia that showed reversal of vitiligo, which is an extremely rare phenomenon, with the objective of probing the probable reasons for this reversal. To the best of our knowledge, there is no study on vitiligo that has reported spontaneous reversal of vitiligo in Saudi Arabia so far. *Materials and Method:* The patient presented to the Family Medicine clinic with a history of restoration of melanin pigment in his lesions after 3 years of the onset of vitiligo. Patients history was taken carefully along with clinical examination, carried out necessary biomedical lab investigations and compiled the data. The data at the time of pigment restoration were compared to the previous data when he developed the lesions. *Result:* The probable reasons for vitiligo reversal could be markedly decreased psychological stress, regular consumption of an antioxidant-rich herbal drink made of curcumin and honey, and dietary switchover to vegetarianism and an alcohol-free lifestyle. *Conclusions:* Curcumin-based herbal remedies could be an alternative option to treat vitiligo. These methods must be further explored through clinical trials as they are safer, easily available, and more affordable.

## 1. Introduction

Vitiligo is a skin disorder characterized by the occurrence of macular hypopigmented lesions on the body due to a lack of melanin production in the affected area as a result of progressive destruction of the melanocytes. Vitiligo has a global prevalence of 0.004% to 9.98% [1], affecting all ethnicities and both genders, and can occur at any age [2]. The lesions can be found on any part of the body. However, they are more commonly present on the face, lips, hands, fingertips, and elbows. These lesions have variable sizes, ranging from a few millimeters to a generalized widespread distribution. Vitiligo with focal lesions is more common; however, in rare cases, the lesions may involve almost 90% of the body surface area, and this type of vitiligo is called universal vitiligo. Vitiligo is usually of two types, the non-segmental and segmental. Non-segmental vitiligo is more common, bilateral, and usually considered to be of autoimmune etiology, whereas segmental vitiligo is uncommon, present on one side of the body, and the proposed mechanism for this type is that the nerve endings of the affected region produce certain chemicals that damage the melanocytes, thereby decreasing melanin production [3]. It is known from the literature that genetic factors account for about 80% and environmental factors account for about 20% of the risk for vitiligo. The melanocytes of vitiligo patients are susceptible to oxidative stress, leading to the release of inflammatory cytokines, which in turn activate innate and adaptive immune responses through activation of autoreactive cytoxic CD8+ cells, which produce molecules such as interferon-γ and other chemokines [4]. A recent study has reported that vitiligo patches may develop as a rare adverse effect of anti-IL-17 therapy, using the ixekizumab drug, which was administered to treat patients with psoriasis [5]. Other researchers have also reported that ixekizumab, an anti-IL drug, could lead to the development of vitiligo patches as an adverse drug effect [6]. Vitiligo is usually asymptomatic but may be associated with symptoms such as itchiness, dryness, and discoloration of hair in the lesions. In due course of time, the lesions progress in size and number. Though the symptoms of vitiligo are not debilitating, patients feel mentally disturbed [7] due to the cosmetic stigma associated with this condition. There is no cure for vitiligo, but certain treatment modalities such as topical cosmetic camouflage creams, steroids, PUVA therapy, narrowband UVB, and depigmenting agents (used in case of vitiligo involving more than 50% area) are available, but the results of these therapies are temporary and do not ensure that the progress of the disease would be halted. Moreover, the side effects of these treatment options and the cost incurred by the patients cannot be overlooked. We came across an interesting case of vitiligo in Saudi Arabia that showed reversal of vitiligo, which is an extremely rare phenomenon and is being presented in this paper with the aim of probing the probable reasons for this reversal. To the best of our knowledge, no study on vitiligo has reported spontaneous reversal of vitiligo in Saudi Arabia.

## 2. Case Presentation

**Figure 1:**
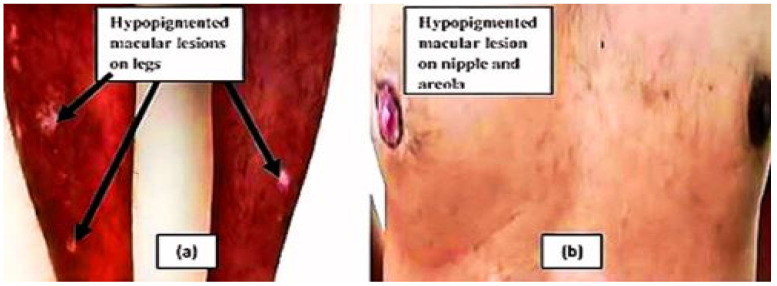
Vitiligo with multiple circular hypopigmented macular lesions on legs (**a**) and depigmented lesions on left nipple and areola (**b**).

A 41-year-old man presented to the Family Medicine clinic of King Khalid University, Saudi Arabia, to discuss certain white spots present on his legs and left nipple. The clinician elicited a history from the patient and learned that he developed three macular lesions on the anterior surface of his right leg about 3 years ago. These lesions were circular in shape, with distinct margins, and initially light in color, with a size of about 5–6 mm, but over a subsequent span of 6 months, the macules became completely colorless and increased in size. Besides this, the patient’s right nipple also became completely depigmented. The patient then got apprehensive and consulted a dermatologist near his home for his skin lesions. The dermatologist examined him, advised certain biochemical investigations (Table 1 and Table 2), and eventually made a diagnosis of vitiligo. The patient said that the dermatologist explained to him that the disorder is not curable and that no treatment was required, as the lesions were present on the covered surface of the body and were likely to remain asymptomatic. However, the patient observed that over the next 7–8 months, the lesions increased in size and number on his right leg, and new lesions appeared on the left leg as well. The lesions were not itchy but were dry, and the hair in the lesions also became depigmented (leukotrichia). The patient, being of the educated class, started to read up on the internet regarding herbal methods for treating vitiligo. From the literature, the patient came to know about the benefits of turmeric and honey for treating skin disorders, because of their antioxidant and anticancer properties. The patient then started to prepare a daily drink of about 200 mL of hot water, to which he added a quarter teaspoon of turmeric powder (curcuma longa) and 1 teaspoon of honey and consumed it after it had cooled down a bit. He completely switched to a vegetarian diet as well. Since then, he has kept following these lifestyle measures to date (approximately the last two years). The patient recently observed that during the last 3 months, some of the lesions on his legs and right nipple had begun to show restoration of pigment. The patient then visited us at the Family Medicine clinic to discuss it. We examined the patient and found that many of the lesions showed repigmentation, filling up to approximately 40–50% of the depigmented area (Figure 1). Upon examination, it was further found that the lesions did not turn erythematous upon rubbing, ruling out the possibility of nevus depigmentosus, nor did the lesions show any change in skin upon diascopy, thereby ruling out the possibility of nevus anemicus. There was no mucosal involvement observed in the patient. The patient did not have a history of any other autoimmune disorders, such as diabetes, hypothyroidism, hyperthyroidism, pernicious anemia, or any systemic or cutaneous disorder. There was no family history of vitiligo. There was no history of chronic drug intake or supplements taken by the patient. The patient was a nonsmoker. He was an occasional alcohol drinker, but has not been consuming alcohol ever since he was diagnosed with vitiligo. When enquired further about the factors that might have led to improvement in lesions, the patient revealed that he had been going through tremendous psychological stress for about 6 years due to family-related issues, but for the last 2 years, during which he developed the lesions, the psychological stress was drastically less. We carried out certain biochemical investigations on the patient and compared them with his previous lab findings (Table 1 and Table 2).

Leukoderma is a condition that resembles vitiligo and that occurs due to chemical-induced depigmentation of skin, especially seen in persons working in dye and chemical industries. However, based upon occupational history, this condition was ruled out in our patient. Pityriasis versicolor is another differential diagnosis of vitiligo that was ruled out in this patient based upon history, morphology of the lesions, and laboratory tests.

## 3. Methods

**Figure 2:**
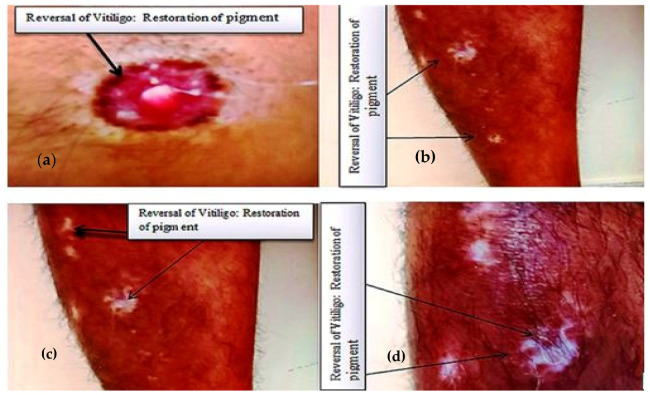
Macular lesions on nipple (**a**) and legs (**b**–**d**) showing restoration of melanin pigment, indicating reversal of vitiligo.

The known case of vitiligo was reported to the Family Medicine clinic of King Khalid University, Abha, Saudi Arabia, in 2022, with a history of restoration of melanin pigment in macular lesions. He was clinically examined and investigated for biochemical profile at Aseer Central Hospital, Abha. The present and previous history, clinical examination findings, and laboratory investigation results were compiled and analyzed. Comparison of the data was done at two time points: when the patient was first diagnosed with vitiligo in 2019 and his recent current status in 2022 (Table 1 and Table 2, Figure 1 and Figure 2). Written informed consent was obtained from the patient for publishing his data, ensuring that his identity would be kept confidential.

## 4. Results

Table 1 shows the epidemiological and clinical profile of the patient, who was a middle-aged man. The patient had switched to a vegetarian and nonalcoholic lifestyle during the course of illness. Table 2 shows the biochemical profile of the patient. It was found that hemoglobin, RBC count, and platelet count was nearly the same in 2019 and 2022, but the WBC count was relatively higher in 2019, when the disorder was first diagnosed. Moreover, the inflammatory markers ESR and hsCRP levels were also abnormally high in 2019. The glycemic profile indicators, fasting blood sugar (FBS), and HbA1c were similar and within the normal reference range at both time points. The lipid profile showed higher total cholesterol and LDL levels and low HDL levels in the patient in 2019; however, the triglyceride was almost similar at both time points. The liver function test (LFT), kidney function test (KFT), uric acid levels, thyroid hormone profile, and 25-hydroxy vitamin D levels were normal at both time points. The KOH microscopy of the lesion that was performed in 2019 was also negative for fungal infection.

## 5. Discussion

**Figure 3:**
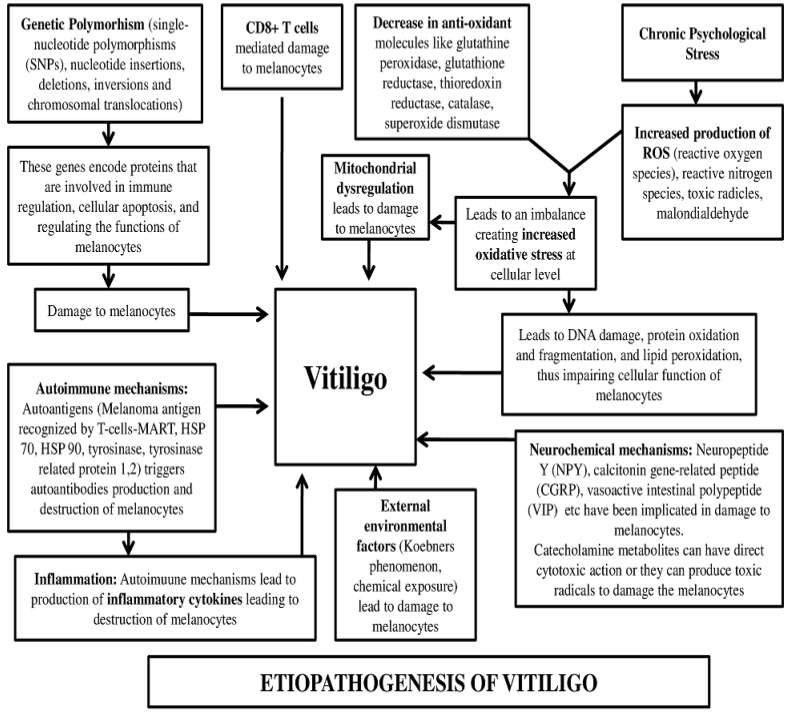
Mechanisms describing the etiopathogenesis of vitiligo. MART (Melanoma Antigen Recognized by T-Cell), HSP (Heat Sock Protein).

In the literature, a conjoint interplay of multiple mechanisms (Figure 3) has been suggested to explain the destruction of melanocytes in vitiligo patients. These include genetic, autoimmune responses, generation of inflammatory mediators, oxidative stress, and melanocyte detachment mechanisms. Some of these theories are discussed in this paper to understand the etiopathogenesis of vitiligo.

Neurochemical theory: Certain molecules, such as neuropeptide Y (NPY), calcitonin gene-related peptide (CGRP), vasoactive intestinal polypeptide (VIP), and catecholamine metabolite, have been implicated in the development of vitiligo [4]. The catecholamine metabolites have direct cytotoxic action on melanocyte in addition to mediating the production of toxic radicles harmful to melanocytes [8,9].

Genetic theory: There is strong evidence from multiple epidemiological studies indicating the importance of genetic factors in the development of vitiligo [10]. Approximately 20% of vitiligo patients have at least one first-degree relative with vitiligo, and the relative risk of vitiligo for first-degree relatives is increased 7- to 10-fold [11]. Genome-wide association studies have identified about 50 different genetic loci that pose a risk for vitiligo [12]. A large number of genes encode proteins that are involved in immune regulation, cellular apoptosis, and the regulation of the functions of melanocytes. Some studies have implicated the role of genetic polymorphism in vitiligo, such as single-nucleotide polymorphisms (SNPs), nucleotide insertions, deletions, inversions, and chromosomal translocations [13,14]

Immunological and inflammatory theories: These theories describe the role of auto antibodies and adaptive and innate immunity as the reason behind the destruction of melanocytes [15]. The role of CD8+ T cells in destroying melanocytes has been explained by certain studies [16,17]. Besides this, a study has described that anti-melanocyte antibody immunoglobulin G mediates human leukocyte antigen-DR (HLA-DR), which subsequently brings about an inflammatory response by cytokine production, leading to destruction of melanocytes [18]. Some studies have described the autoantibodies that recognize certain antigens on melanocytes. Some of these auto antigens are HSP 70, tyrosinase-related proteins 1 and 2, and tyrosinase [19,20].

Oxidative stress theories: Researchers have proposed the role of oxidative stress in vitiligo that leads to damage to melanocytes [21] arising from an imbalance between oxidative stress and antioxidant mechanisms. The reactive oxygen species (ROS) destroys the melanocytes, producing hypopigmented lesions in vitiligo patients. Oxidative stress also leads to mitochondrial dysfunctions, leading to damage to melanocytes [22].

The authors have reported a case of vitiligo in Saudi Arabia that has shown spontaneous reversal of vitiligo manifested in the form of repigmentation of macular lesions. It is an extremely rare phenomenon, as the majority of vitiligo patients show a progressive pattern. Though vitiligo is a benign condition, such patients often face social stigma, leading to low confidence. Some patients do seek pharmacological treatment using steroids, PUVA, and narrowband UVB therapy. Though pigmentation begins to restore after a few months with these treatment options, there is an associated risk of the development of skin cancers. Even if the pigment restores, the condition may progress or relapse. Therefore, alternative safer methods must be explored by the researchers, especially using herbal or natural methods that could be more affordable and easily available. In our study, the patient was consuming a herbal drink comprising turmeric powder (botanical name: curcuma longa; contains active principle curcumin) and honey. Turmeric is known as the golden spice, and it contains a polyphenol called curcumin [23]. Curcumin is well known for its medicinal properties owing to its anti-inflammatory, antioxidant, anticancer, antiviral, and antibacterial actions [24]. There has been vast research on its medicinal properties to treat several diseases. A study by Asawononda et al. showed that patients with vitiligo who were treated with narrowband UVB phototherapy in combination with topical tetrahydrocurcuminoid cream showed better repigmentation of vitiligo lesions in comparison to those treated with narrowband UVB monotherapy [25]. A study by Irshad et al. showed that depigmented skin in vitiligo showed partial repigmentation after 4 months of natural herbal treatment using honey [26]. Honey contains molecules such as proteins, fats, carbohydrates, polyphenols, and vitamin C. Contents of honey have been shown to accelerate pigmentation [27]. Besides, carbohydrate, fat, glucose, sucrose, and honey contain more than 180 substances that depict their complex nature, to be used as antibacterial, antiparasitic, antiviral, anti-inflammatory, antioxidant, antitumor, and anti-mutagenic effects [26]. Facial vitiligo was found to be successfully treated by Zouhiretal in his study using a combination of honey, allium cepa, and avena sativa [28]. Since the role of oxidative stress and inflammatory molecules such as cytokines has been already discussed above, it is logical to think that consumption of food items that have antioxidant and anti-inflammatory properties is likely to benefit vitiligo patients. The abnormally high ESR and hsCRP levels came down drastically in 2022 (Table 2) when the patient showed reversal of vitiligo. This could be the possible evidence that curcumin and honey would have decreased his ongoing inflammation and oxidative stress. A case control study by Reza et al. also showed that vitiligo patients have high hsCRP levels [29], as C-reactive protein is an acute phase reactant protein that is secreted in blood in response to inflammatory cytokines such as IL-6. A study by Shankar et al. also reported high ESR levels in vitiligo patients [30].

Since immunological theory and the role of CD8+ T cells in the etiopathogenesis of vitiligo has been implicated in the literature, it is rational to check for a patient’s WBC count. This patient had a higher WBC count when he was diagnosed with vitiligo in 2019, but the WBC count relatively decreased after he consumed a herbal drink and adopted other lifestyle measures.

Moreover, the patient switched to a vegetarian diet and an alcohol-free lifestyle, which might have further decreased his oxidative stress in the body. Studies have found that a vegetarian diet decreases oxidative stress [31]. A study conducted by Das et al. reported that ethanol metabolism is directly involved in the production of reactive oxygen species (ROS) and reactive nitrogen producing species (RNS), amounting to oxidative stress [32].

The patient had a strong history of chronic psychological stress for 6 years, which could have possibly been the main trigger for the onset of vitiligo. Mental stress manifests at the cellular level in the form of oxidative stress and inflammatory reactions. The patient reported reversal of vitiligo when there was a decrease in mental stress levels for the past two years. Studies in the literature have also reported that emotional or stressful events seem to play a pivotal role in vitiligo onset or exacerbation [7,8].

In our study, the patient had relatively higher total cholesterol, LDL, and lower HDL when he was first diagnosed with vitiligo, which was similar to the findings reported by Zeinab et al. [33] in vitiligo patients. The higher total cholesterol in our patient could be due to his nonvegetarian food habits until he was diagnosed with vitiligo.

Normal glycemic profile (FBS, HbA1c), thyroid profile, vitamin D, LFT, and KFT levels (Table 2) indicated that the patient had no other autoimmune systemic involvement.

## 6. Conclusions

In our study, we reported a case of vitiligo in Saudi Arabia that has shown spontaneous reversal of vitiligo with repigmentation of macular lesions. This is an extremely rare phenomenon. The probable reasons for the same could be markedly decreased psychological stress, regular consumption of an antioxidant-rich herbal drink made of curcumin and honey, and dietary switchover to vegetarianism and an alcohol-free lifestyle. Based upon the findings of this study, the authors recommend that in the future more clinical trials should be taken up on patients with vitiligo using such curcumin-based herbal remedies to explore their effect clinically and on biochemical parameters, as herbal methods are safer, easily available, and affordable for most patients. Such studies will confirm our findings, which at present remains a hypothesis. So far, very few studies have explored the role of curcumin and honey in vitiligo. We could report our findings in a single patient only because spontaneous reversal of vitiligo is a very rare phenomenon. The use of curcumin in the real setting seems promising but deserves further studies to definitely enter into the clinical armamentarium.

### Implications of the Study

Although many regimes have been used in vitiligo treatment, there is still no permanent cure for this disease and the high cost also involved. Other adverse effects of drugs used may worsen the outcomes. Very few studies have explored the effective role of curcumin-based herbal remedies, and more study designs need to be established for herbal-based treatment as cost-effective management with fewer side effects. In our case report, we aimed to show the effects of herbal-based treatment.

Wearing a face mask became mandatory during the COVID-19 pandemic and could lead to vitiligo on the face, a condition seen in the Koebner phenomenon [34]. This curcumin-based remedy may be helpful in arresting the progress of the disease in the mass and marginalized population as a cost-effective treatment. The side effects of immunotherapies may manifest with cutaneous toxicity and cause confetti-like depigmentation of vitiligo in melanoma-treated patient [35]. Curcumin can be used in the treatment of melanoma, as many studies suggest that curcumin is a powerful antioxidant with anticancer properties. More scientific evidence needs to be gathered to support the effectiveness of other supplements, such polypodium leucotomos, which is a fern used in the treatment of psoriasis, atopic dermatitis, vitiligo, etc. Further research should focus on the synergistic action of polypodium leucotomos and curcumin together to gain a better understanding of the mechanism of action at the molecular level.

## Figures and Tables

**Table 1:** Epidemiological and clinical features of the patient with vitiligo.

Variable	Previous Data (When Vitiligo Was First Diagnosed in 2019)	Current Data (When Vitiligo Lesions Showed Reversal in 2022)
Age (years)	38	41
Weight (kg)	70	75
BMI (kg/m^2^)	23.7	25.4
Smoker	No	No
Alcoholic	Occasional	No
Dietary habit	Nonvegetarian	Vegetarian
% of body surface area covered with macules	<10%	<10%
Duration of vitiligo	6 months	3 years
Leukotrichia	Present	Present
Koebner phenomenon	Absent	Absent
Mucosal involvement	None	None
Diascopy findings	Not available	Uniformly depigmented lesions observed with sharp demarcated margins; no color change observed on diascopy, ruling out nevus depigmentosus
On rubbing the lesions	No erythema (nevus anemicus ruled out)	No erythema (nevus anemicus ruled out)
Any associated cutaneous disorder such as alopecia areata, halo nevus, lichen planus, plaque psoriasis, alopecia areata, and ichthyosis vulgaris	None	None
Any associated systemic disorder such as diabetes, hypothyroidism, hyperthyroidism, and pernicious anemia	None	None
Blood pressure; SBP/DBP (mm Hg)	124/84	118/82
Blood group	B+	B+

BMI (Body Mass Index), SBP (Systolic Blood Pressure), DBP (Diastolic Blood Pressure).

**Table 2:** Biochemical features of the patient with vitiligo.

Variable	Previous Data (When Vitiligo Was First Diagnosed in 2019)	Current Data (When Vitiligo Lesions Showed Reversal in 2022)
Hemoglobin (g%)	14.5	15.2
RBC count (million cells/cu mm)	4.8	5.1
WBC count (/cu mm)	9320	7250
Platelet count (/cu mm)	250,000	285,000
ESR (mm/hr)	22	5
hsCRP (mg/L)	9.2	2
FBS (mg/dL)	102	85
HbA1c (%)	5.2	4.8
Total cholesterol (mg/dL)	250	161
Triglyceride (mg/dL)	130	125
LDL (mg/dL)	105	90
HDL (mg/dL)	38	51
LFT	Normal	Normal
KFT	Normal	Normal
Uric acid (mg/dL)	5.4	5.2
Total T3 (ng/mL)	1.2	1.4
Total T4 (µg/dL)	7.5	8
TSH (µIU/mL)	3.5	3.2
25-hydroxy vitamin D (ng/mL)	25	28
KOH microscopy	Negative	Not done

WBC (White Blood Cell), ESR (Erythrocyte Sedimentation Rate), hsCRP (high sensitivity C- Reactive Protein), FBS (Fasting Blood Sugar), HbA1c (Hemoglobin A1c), LDL (Low Density Lipoprotein), HDL (High Density Lipoprotein), LFT (Liver Function Test), KFT (Kidney Function Test), T3 (Triiodothyronine), T4 (Tetraiodothyronine), TSH (Thyroid Stimulating Hormone), KOH (Potassium Hydroxide).

## Data Availability

Not applicable.
